# Tapwater Exposures, Effects Potential, and Residential Risk Management in Northern Plains Nations

**DOI:** 10.1021/acsestwater.2c00293

**Published:** 2022-09-26

**Authors:** Paul M. Bradley, Kristin M. Romanok, Kelly L. Smalling, Michael J. Focazio, Robert Charboneau, Christine Marie George, Ana Navas-Acien, Marcia O’Leary, Reno Red Cloud, Tracy Zacher, Sara E. Breitmeyer, Mary C. Cardon, Christa K. Cuny, Guthrie Ducheneaux, Kendra Enright, Nicola Evans, James L. Gray, David E. Harvey, Michelle L. Hladik, Leslie K. Kanagy, Keith A. Loftin, R. Blaine McCleskey, Elizabeth K. Medlock-Kakaley, Shannon M. Meppelink, Joshua F. Valder, Christopher P. Weis

**Affiliations:** † U.S. Geological Survey, Columbia, South Carolina 29210, United States; ‡ U.S. Geological Survey, Lawrenceville, New Jersey 08648, United States; § U.S. Geological Survey, Reston, Virginia 20192, United States; ∥ Spirit Lake Tribe Office of Environmental Health, Fort Totten, North Dakota 58335, United States; ⊥ Johns Hopkins Bloomberg School of Public Health, Baltimore, Maryland 21205, United States; # Columbia University Mailman School of Public Health, New York, New York 10032, United States; ¶ Missouri Breaks Industries Research Inc., Eagle Butte, South Dakota 57625, United States; ∇ Oglala Sioux Tribe Natural Resources Regulatory Agency, Pine Ridge, South Dakota 57770, United States; ○ U.S. Environmental Protection Agency, Durham, North Carolina 27709, United States; ⧫ U.S. Geological Survey, Lakewood, Colorado 80228-3742, United States; †† Indian Health Service/HHS, Rockville, Maryland 20857, United States; ‡‡ U.S. Geological Survey, Sacramento, California 95819, United States; §§ U.S. Geological Survey, Lawrence, Kansas 66049, United States; ∥∥ U.S. Geological Survey, Iowa City, Iowa 52240, United States; ⊥⊥ U.S. Geological Survey, Boulder, Colorado 80303, United States; ## U.S. Geological Survey, Rapid City, South Dakota 57702, United States; ¶¶ National Institute of Environmental Health Sciences/NIH, Bethesda, Maryland 20814, United States

**Keywords:** tapwater, arsenic, organics, inorganics, human health, private wells, underserved communities

## Abstract

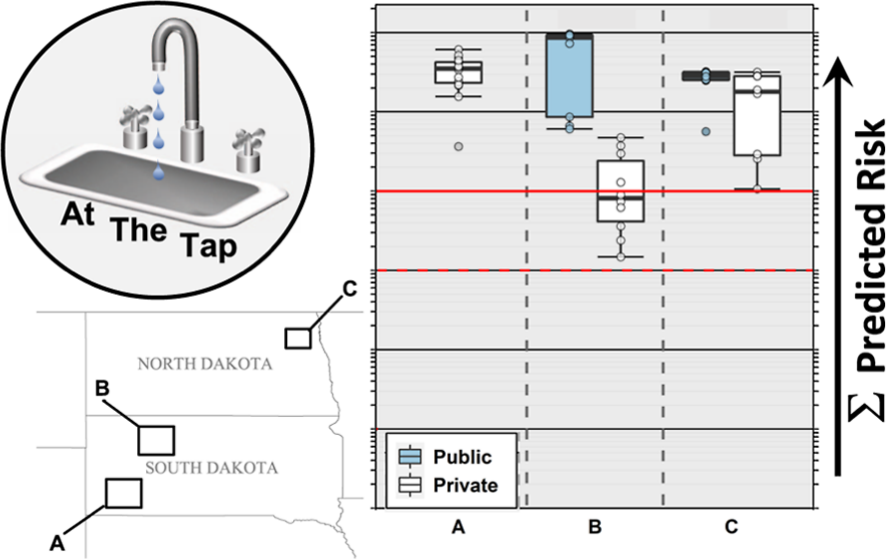

In the United States (US), private-supply tapwater (TW) is rarely monitored. This data gap undermines individual/community risk-management decision-making, leading to an increased probability of unrecognized contaminant exposures in rural and remote locations that rely on private wells. We assessed point-of-use (POU) TW in three northern plains Tribal Nations, where ongoing TW arsenic (As) interventions include expansion of small community water systems and POU adsorptive-media treatment for Strong Heart Water Study participants. Samples from 34 private-well and 22 public-supply sites were analyzed for 476 organics, 34 inorganics, and 3 in vitro bioactivities. 63 organics and 30 inorganics were detected. Arsenic, uranium (U), and lead (Pb) were detected in 54%, 43%, and 20% of samples, respectively. Concentrations equivalent to public-supply maximum contaminant level(s) (MCL) were exceeded only in untreated private-well samples (As 47%, U 3%). Precautionary health-based screening levels were exceeded frequently, due to inorganics in private supplies and chlorine-based disinfection byproducts in public supplies. The results indicate that simultaneous exposures to co-occurring TW contaminants are common, warranting consideration of expanded source, point-of-entry, or POU treatment(s). This study illustrates the importance of increased monitoring of private-well TW, employing a broad, environmentally informative analytical scope, to reduce the risks of unrecognized contaminant exposures.

## Abstract 2

Elevated human risk from co-occurring inorganic and organic contaminants observed in public and private tapwater in United States tribal lands.

## 1 Introduction

Water is life (Mní wičhóni in Lakota). The quality and sustainability of drinking water are growing challenges in the United States (US) and world wide due to, among other reasons, increasing water demands and drinking-water source contamination.^1−3^ US public, private, and bottled drinking-water supplies share many anthropogenic-contaminant (i.e., human-generated/-driven) concerns, because most of the 350,000 chemicals estimated to be in commercial use globally^4^ and, by extension, potentially in drinking-water source waters^5,6^ are not currently regulated or systematically monitored in public-supply tapwater (TW)^7,8^ or in bottled drinking water.^9^ Note, the U.S. Environmental Protection Agency (EPA) does not regulate private wells nor does it provide recommended criteria or standards for individual wells.^10^ In contrast, many water-borne pathogens (e.g., *Cryptosporidium*) and naturally occurring contaminants (e.g., arsenic, As) are actively regulated in US public and bottled-water supplies.^7−9^

The potential for unrecognized contaminant exposures and adverse health effects is notably elevated for private and small community drinking-water supplies in rural and remote areas,^11,12^ due to differences in regulation, technical expertise, natural/economic resources, and associated risk-management options.^13−15^ Although some geogenic groundwater contaminants, such as As, exhibit broadly predictive geospatial patterns,^16,17^ individual point-of-use (POU) TW concentrations reflect multiple factors including well-screen/open-hole depth, geologic stratigraphy, and spatial/temporal variability in water levels and redox conditions and, consequently, can differ substantially and unpredictably from well to well.^18,19^ Further, As and several other contaminants are imperceptible (tasteless and odorless) without TW testing.^11,12,20^

Prior studies have documented a range of contaminant concerns in unregulated drinking water.^12,20,21^ Due to high analytical costs, common-place conflation of organoleptic quality with safety, and other socioeconomic factors, private-well water-quality data remain scarce and, where available, are typically limited to a few targeted contaminants.^20−23^ Similarly, small rural water systems are often concerns for various socioeconomic reasons, including financial limitations of smaller and lower-income populations.^24−27^ The elevated probability of unrecognized exposures has prompted calls for universal private-well As testing,^23,28^ but, acknowledging the increasingly human-impacted water cycle,^29,30^ broader characterization of private-well and rural-water-system contaminant exposures is needed.^31^

The Strong Heart Study (SHS) is an ongoing population-based prospective-cohort study of cardiovascular disease and associated risk factors among American Indian (AI) adults in participating Tribal Nations in Arizona, North Dakota, Oklahoma, and South Dakota.^32^ Groundwater As varies regionally across the US, with elevated concentrations predicted and observed^16,17,33,34^ throughout SHS areas. SHS research has associated low to moderate drinking-water As exposures with lung, prostate, and pancreatic cancer,^35^ cardiovascular disease,^36^ kidney disease,^37^ lung disease,^38^ and type-2 diabetes outcomes.^39,40^ Efforts to manage As-related health risks in SHS-area TW include the development/expansion of rural water systems and the Strong Heart Water Study (SHWS), a participatory randomized controlled intervention to reduce As exposures in remote residences in North and South Dakota using undersink adsorptive-media POU treatment.^41,42^

The U.S. Geological Survey (USGS) collaborates with EPA, the National Institute of Environmental Health Science (NIEHS), Food and Drug Administration (FDA), Tribal Nations, universities, utilities, communities, and others to inform drinking-water exposure and water-supply data gaps by assessing TW inorganic/organic contaminant mixtures and associated distal (e.g., ambient source water) and proximal (e.g., premise plumbing and POU treatment) drivers in a range of socioeconomic and source-water vulnerability settings across the US.^29,30,43,44^ As part of that ongoing effort, we assessed exposures to a broad suite of potential inorganic and organic TW contaminants in 56 homes in three areas of North Dakota and South Dakota in 2019 to provide insight into cumulative contaminant risk to human health^45−47^ of private-well and small community public-supply TW in this region, to assess the utility of broader TW contaminant assessments for identifying additional risk management efficiencies in areas undergoing drinking-water interventions for As, and to expand the national perspective on inorganic and organic contaminant exposures at the TW point of use by maintaining the same general sampling protocol and analytical toolbox employed in previous studies.^29,30,43,44^

For this study, TW exposure was operationally represented as concentrations of 476 organics, 34 inorganics, and 3 bioactivity indicators in residential and community POU TW samples. Potential human-health risks of individual and aggregate TW exposures were explored based on cumulative detections/concentrations of designed-bioactive chemicals (e.g., pesticides and pharmaceuticals),^5^ as well as the cumulative exposure-activity ratio(s) (Σ_EAR_)^48^ and hazard indices (HI)^49,50^ of cumulative benchmark-based toxicity quotient(s) (TQ) (Σ_TQ_).^51^ In line with previous results by this research group and others, simultaneous TW exposures to multiple inorganic and organic constituents of potential human-health interest were hypothesized to occur in both private- and public-supply samples.^12,21,29,30^

## 2 Methods

### 2.1 Site Selection and Sample Collection

**Figure 1:**
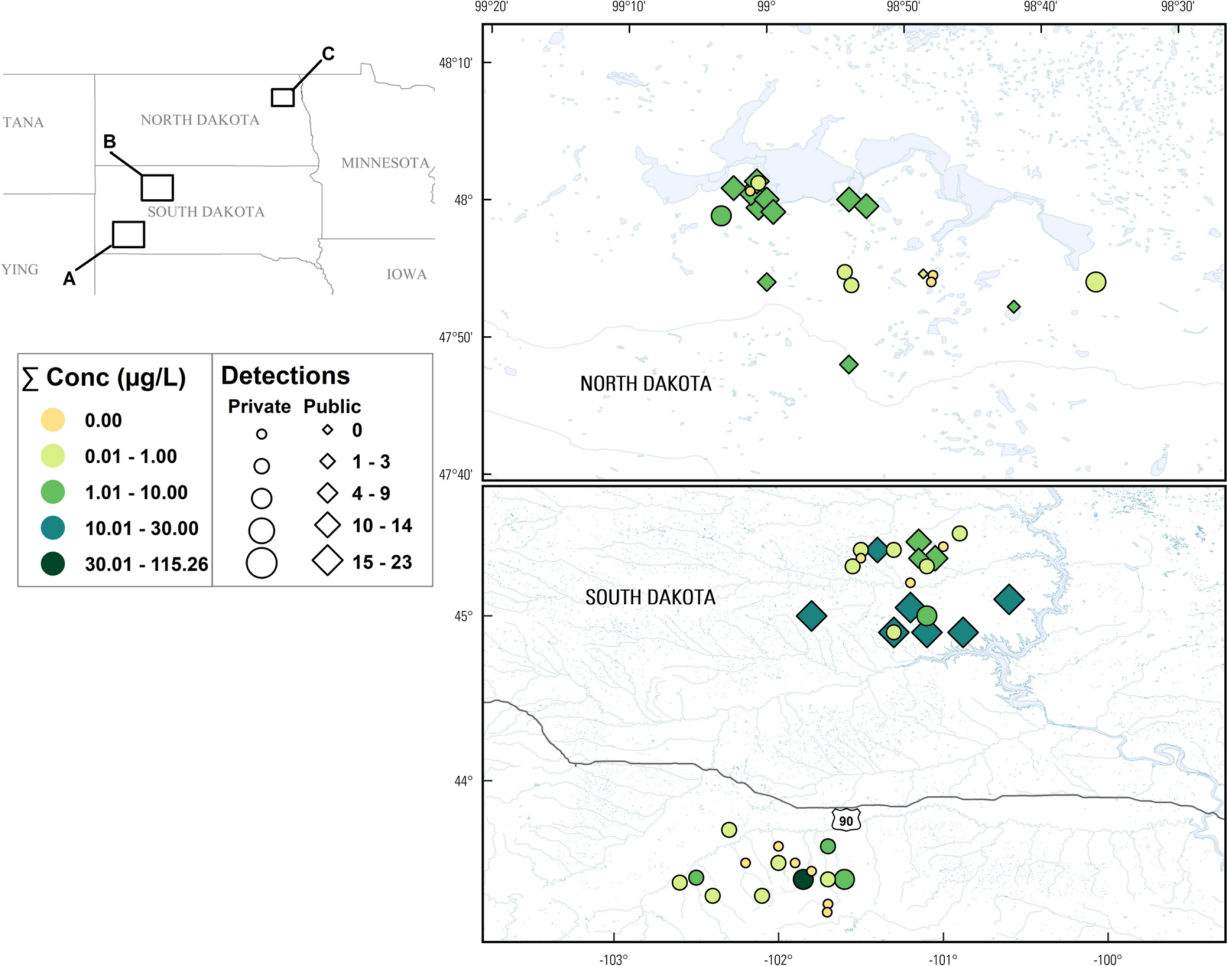
Cumulative (sum of all detected) concentrations (μg L^–1^) and numbers of organic compounds in samples of public-supply (diamonds, ◆) and private-supply (circles, ●) TW collected during 2019 in North Dakota and South Dakota. Sample locations are anonymized.

Sample locations were selected from community volunteers to provide broad spatial coverage of private-/public-supply TW in three Tribal areas in North Dakota and South Dakota,^41^ with 16 private; 10 private/10 public; and 8 private/12 public TW locations from communities A, B, and C, respectively (Figures 1 and S1). All community A locations were SHWS participants, for which the inclusion criterion was greater than maximum contaminant level(s) (MCL) baseline TW As concentrations. In this study, all private-supply and 15 public-supply TW samples were groundwater sourced. In community B, three and seven public-supply sample locations were connected to groundwater-sourced and surface-water-sourced (Missouri River) water systems, respectively. Community C public-supply samples were groundwater sourced, with 11 samples from the primary, multiwell water system and one sample from a small (65 people) system. In general, TW samples were collected from untreated kitchen faucets, except for four (three private, one public) community B samples collected from POU-treatment taps (denoted POU-AF in Supporting Information Tables) at the participants’ request. One other TW-sample location (community B) had a point-of-entry water-softener system (POE-S in Supporting Information Tables).

Taps (cold water) were sampled once each from September to November 2019. Samples were collected at the participant’s convenience throughout the day, without precleaning, screen removal, or Lead and Copper Rule stagnant-sample protocols.^52,53^ Complete sampling details are provided elsewhere.^54−56^

### 2.2 Methods and Quality Assurance

Briefly, TW samples were analyzed by USGS using seven organic (6 classes; 476 total/468 unique analytes), five inorganic (34 ions/trace elements), and two field (3 parameters) methods (Table S2) and by USEPA using three in vitro bioassay (ER, AR, and GR) methods, as discussed^29,30,43,54^ and described in detail previously.^57−74^ Organic analytes included cyanotoxin, disinfection byproduct(s) (DBP), pesticide, per/polyfluoroalkyl substance(s) (PFAS), volatile organic compound(s) (VOC), and pharmaceutical classes; additional method details are in the Supporting Information. All results are in Tables S3–S4 and S8 and in Romanok et al.^55,56^ Quantitative (≥limit of quantitation, ≥LOQ) and semiquantitative (between LOQ and long-term method detection limit, MDL^75,76^) results were treated as detections.^75,77,78^ Quality-assurance/quality-control included analyses of six field blanks, as well as laboratory blanks, spikes, and stable-isotope surrogates. Only bromide was detected in inorganic blanks at concentrations in the range observed in TW samples; results were censored at the maximum blank concentration (0.14 mg L^–1^), as footnoted (Tables S3 and S5). Among detected organics, only ethyl acetate (0.14 μg L^–1^) was detected in a blank (1) in the concentration range observed in TW samples; results were censored at two times the maximum blank concentration (0.28 μg L^–1^), as footnoted (Tables S4 and S6). The median surrogate recovery (Table S7) was 99.9% (interquartile range: 89–110%)

### 2.3 Statistics and Risk Assessments

Differences between TW-sample groups were assessed by nonparametric one-way PERMANOVA (*n* = 9999 permutations) on Euclidean distance (Paleontological Statistics, PAST, vers. 4.03).^79^ Relations between detected TW contaminants were assessed by Spearman rank-order correlation (ρ) and permuted (*n* = 9999) probabilities (PAST, vers. 4.03).^79^

Screening-level assessments^49,50^ of potential cumulative effects were based on the cumulative exposure-activity ratio(s) (Σ_EAR_)^48^ and HI^49,50,80^ of cumulative benchmark-based TQ (Σ_TQ_),^51^ as described.^29^ ToxEval version 1.2.0^81^ of R^82^ was used to sum (noninteractive concentration addition model^83−85^) individual ToxCast-based^86,87^ exposure activity ratios (EAR) or benchmark-based TQ, respectively. For the latter, the most protective human-health benchmark (i.e., lowest benchmark concentration) among MCL goal(s) (MCLG),^7,88^ WHO guideline values (GV) and provisional GV,^89^ USGS Health-Based Screening Level (HBSL),^90^ and state drinking-water MCL or health advisories (DWHA) was used. MCLG values of zero (i.e., no identified safe-exposure level for sensitive subpopulations, including infants, children, the elderly, and those with compromised immune systems and chronic diseases^7,91^) were set equal to the method reporting limit or 1 μg L^–1^ for lead (Pb).^92^ Cumulative EAR (Σ_EAR_) and TQ (Σ_TQ_) results, ToxCast exclusions, and health-based benchmarks are summarized in Tables S9–S13 (additional details in Supporting Information).

## 3 Results and Discussion

**Figure 2:**
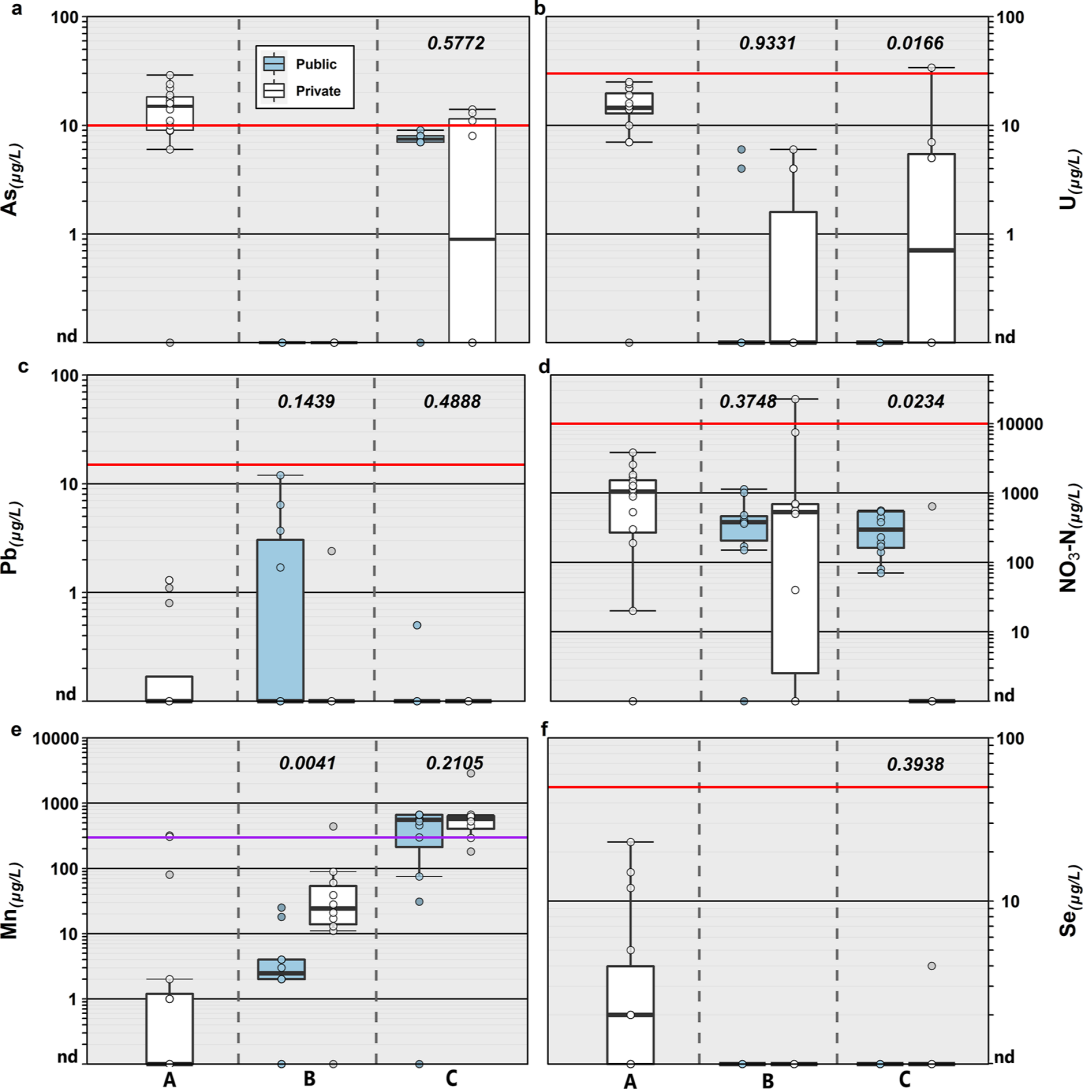
Concentrations (circles, ●) of select inorganics detected during 2019 in North Dakota and South Dakota in public-supply (shaded) and private-supply (unshaded) TW samples within three study areas. Solid red lines indicate public-supply enforceable maximum contaminant level(s) (MCL) or technology treatment action level (Pb). MCL goals (MCLG) for As, U, and Pb are zero. The solid purple line indicates the Mn lifetime drinking water health advisory. Boxes, centerlines, and whiskers indicate interquartile range, median, and 5th and 95th percentiles, respectively. Numbers above each boxplot pair indicate the permuted probability that the centroids and dispersions are the same (PERMANOVA; 9999 permutations). “nd” indicates not detected.

**Figure 3:**
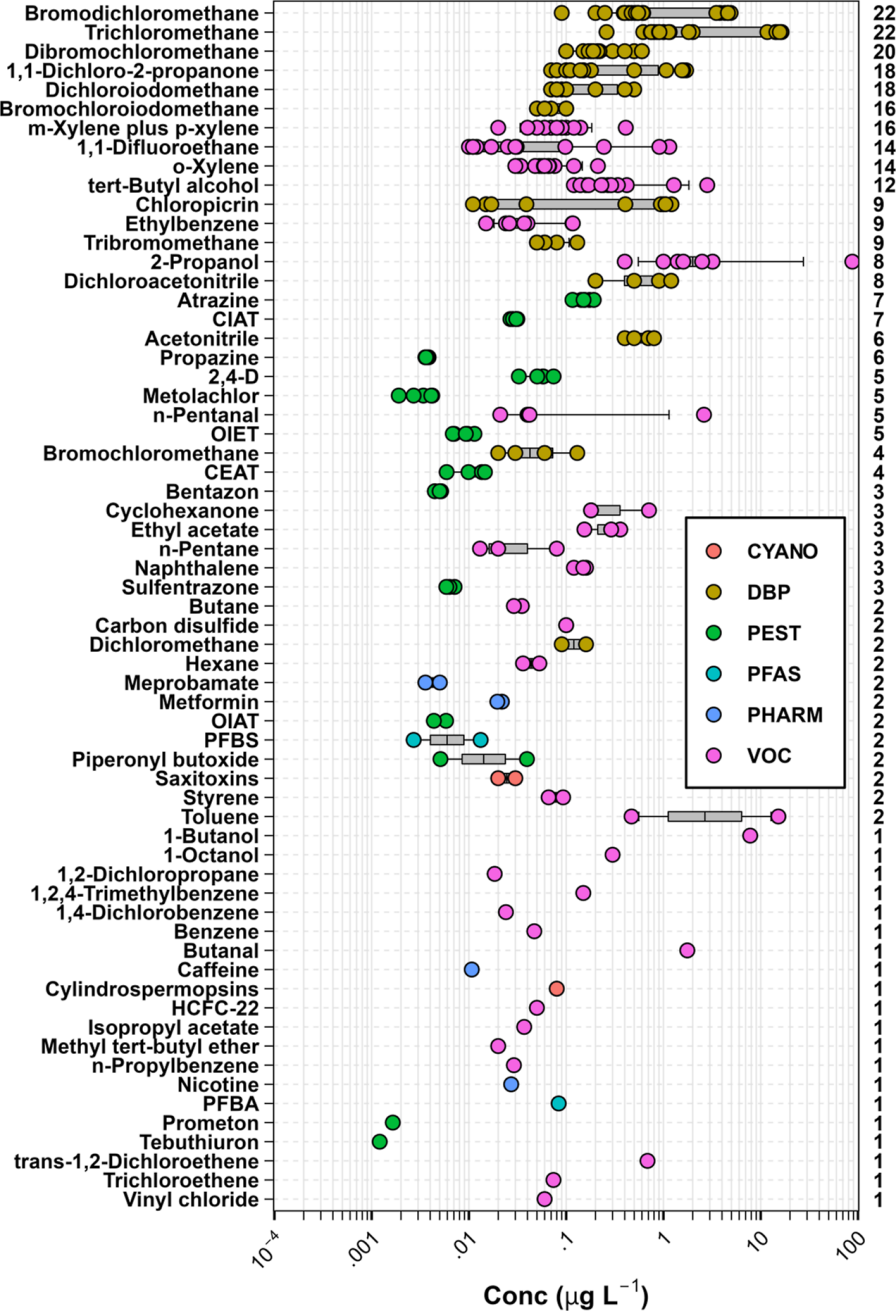
Detected concentrations (μg L^–1^) and the number of sites (right axis) for 63 organic analytes (left axis, in order of decreasing total detections) detected in TW samples collected during 2019 in North Dakota and South Dakota. Circles are data for individual samples. Boxes, centerlines, and whiskers indicate interquartile range, median, and 5th and 95th percentiles, respectively.

**Figure 4:**
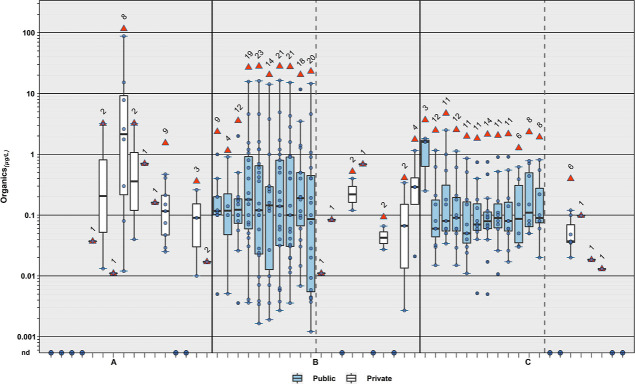
Individual (circles, ●) and cumulative (sum of all detected; red triangles, ▲) concentrations of organic analytes detected in public-supply (shaded) and private-supply (unshaded) TW samples collected during 2019 in North Dakota and South Dakota. Boxes, centerlines, and whiskers indicate interquartile range, median, and 5th and 95th percentiles, respectively. Numbers above each boxplot indicate total detected organic analytes. “nd” indicates not detected.

Regulated and unregulated chemicals (inorganic, organic) were detected in TW samples in all three study areas (Tables 1, S3, and S4; Figures 1–4), with two or more detections of human-health interest commonly observed per sample. 63 (14%) organic and 30 (91%) inorganic analytes were detected. In this discussion, the enforceable EPA MCL or, for Pb, action level (AL) for technology treatment is provided from a regulatory perspective, but the organ/organism-level human-health effects of individual contaminant exposures are contextualized based on MCLG and human-health advisories, for three reasons. First, EPA MCL and AL are enforceable in public supplies^7,8^ but not in private supplies.^10^ Second, MCL and AL are set as close to MCLG values as feasible but are often higher to reflect technical and financial constraints of drinking-water monitoring and treatment.^91^ Last, MCLG and health advisory values generally include a margin of exposure to provide a safety threshold, in the case of MCLG defined as the concentration below which there is no known risk to the health of presumptive “most vulnerable” (e.g., infants, children, pregnant women, elderly, and immune-compromised) subpopulations.^91^

### 3.1 TW Arsenic, Uranium, and Lead

Consistent with previous reports,^33,34,41,93^ TW exposures to As, U, and Pb, which have no known safe level of drinking-water exposure for vulnerable sub-populations (MCLG zero), were widely observed (54, 43, and 20% of samples, respectively) in the three study areas in private- and public-supply locations (Figure 2).

In line with earlier SHWS findings,^41^ the redox-reactive geogenic contaminant As was consistently detected (MCLG exceedance) in communities A and C (94 and 75% of samples, respectively), but not in community B. Consistent with the SHWS inclusion criterion, As was detected in all but one community A sample, near (9 μg L^–1^ in three samples) or above (11 samples) MCL-equivalent concentrations in 88% (14/16) of samples. In community C, As was detected in 50% (4/8) and 92% (11/12) of private- and public-supply samples, respectively, but MCL-equivalent exceedances occurred only in three private-well locations. Overall, the results support ongoing expansions/connections to public supplies as an effective approach to limit TW As-exposures to <MCL concentrations in these communities and POU/POE treatment^41,42^ in private-supply locations where a public-supply connection is not feasible. The results emphasize the potential for unrecognized exposures to contaminants, including contaminants of human-health concern, in unregulated/unmonitored private supplies and support private-well As monitoring.^23,28,33^ The results also support the potential use of POU/POE treatment at public-supply locations with detectable As, as an additional As removal step. While As-associated Safe Drinking Water Information System (SDWIS) violations,^94^ As concentrations, and urinary As levels^95,96^ associated with public supplies have decreased substantially since the MCL was lowered, no safe level of TW As exposure is recognized for vulnerable subpopulations.^97^ Drinking water As exposure is associated with various cancers,^98,99^ organ-system toxicity,^98^ cardiovascular diseases,^100,101^ diabetes,^100,101^ adverse pregnancy outcomes,^102^ and mortality.^102,103^ Adverse health associations with <MCL As exposures^35,98,100,104^ have prompted a 5 μg L^–1^ MCL in some US states (e.g., New Jersey and New Hampshire^105,106^). Every private-well sample with detectable As and 92% of community C public-supply samples exceeded 5 μg L^–1^.

Also, consistent with previous findings,^34,93^ the redox-reactive geogenic radionuclide U was detected (MCLG exceedance) in all three communities (A: 94%, B: 25%, and C: 20%). Drinking-water U is associated with nephrotoxicity^107,108^ and osteotoxicity^109^ in humans, inhibition of DNA-repair mechanisms in human embryonic kidney 293 (HEK293) cells,^110^ and estrogen-receptor effects in mice.^111^ The MCL-equivalent concentration was exceeded only in one private-well location. Community C U exposures differed (PERMANOVA, *p* = 0.013) between private- (4 detections) and public-supply (no detections) locations. No difference was apparent in community B (*p* = 0.93), with 2–3 detections (≤6 μg L^–1^) each in private-well and public-supply samples. Other widely documented, drinking-water radionuclides (e.g., radium and radon)^6,21^ not assessed herein should be included in future assessments.

As and U co-occurred in 16 (42%) of the 38 samples in which either was detected and were moderately positively correlated (Spearman ρ = 0.48, permutation [*n* = 9999] *p* = 0.0002). Because groundwater As and U are favored under reducing and oxidizing conditions, respectively, frequent co-occurrence in SHWS well water suggests redox heterogeneity is common^34^ or redox-independent mechanisms (e.g., pH/alkalinity-driven solubility) are important.^93^ Elevated iron (Fe) and manganese (Mn) concentrations in oxic TW herein support the former. A moderate correlation (ρ ≥ 0.42; *p* < 0.0001) between U and nitrate (NO_3_) was reported^112^ for the High Plains aquifer, the northern extension of which underlies community A; a weaker correlation (ρ ≥ 0.27; *p* < 0.042) was observed herein.

Pb was detected sporadically (20% of samples) in private- and public-supply locations in all communities but did not exceed the AL-equivalent concentration. Elevated drinking-water Pb-exposures primarily are associated with neurocognitive impairment in infants and children.^92,113^ The American Academy of Pediatrics^92^ recommends that drinking-water Pb not exceed 1 μg L^–1^, a common MDL for US public-supply compliance monitoring.^52^ Drinking-water Pb is attributed primarily to premise-plumbing and distribution-infrastructure materials^113^ that predate the 1986 SDWA Amendments.^114^ In this study, no difference in Pb detections (*p* ≥ 0.11) between private- and public-supplies and no systematic Pb detections within a given public-supply system support premise plumbing as the probable source. Importantly, the current results likely underestimate TW Pb occurrence and concentrations in the study area, because flushing effectively decreases plumbing-derived contaminant concentrations,^113,115^ and same-day prior TW use was common in this study.

### 3.2 TW Nitrate

TW nitrate–nitrogen (NO_3_–N) concentrations were generally low (median: 0.4 mg L^–1^) in the study area, with elevated (>2 mg L^–1^) concentrations, including one MCL-equivalent exceedance (Table 1), observed infrequently (4 samples) and only in private-well locations. No co-contaminants (e.g., human-use pharmaceuticals) indicative of human-waste sources (e.g., septic systems) were detected in elevated NO_3_–N samples, indicating other sources, such as inorganic/organic crop fertilizers or animal wastes, as possible contributors to elevated TW NO_3_–N concentrations; the lack of detectable pesticides in the four TW samples with >2 mg L^–1^ NO_3_–N points toward the latter. The NO_3_–N MCLG was established to protect against bottle-fed infant (<6 months) methemoglobinemia.^7^ Emerging evidence associates <MCL NO_3_–N concentrations with other adverse health outcomes,^116^ including cancer,^117^ thyroid disease,^118^ and neural tube defects.^119^

**Table 1:** Number of Exceedances of Maximum Contaminant Level (MCL) Concentrations (MCL-Equivalent for Private Well Locations) or Listed Health GVs Observed in TW Samples^a^

				public supply		private wells		
class	constituent	guidance^a^	value	community B (*N* = 10)	community C (*N* = 12)	community A (*N* = 16)	community B (*N* = 10)	community C (*N* = 8)
inorganic	arsenic	MCL	10 μg/L	0	0	11	0	3
		MCLG	0 μg/L	0	11	15	0	4
	lead	AL	15 μg/L	0	0	0	0	0
		MCLG	0 μg/L	4	2	4	1	0
	manganese	DWHA	300 μg/L	0	9	2	1	7
	nitrate	MCL(MCLG)	10 mg/L	0	0	0	1	0
	selenium	MCL(MCLG)	50 μg/L	0	0	0	0	0
		Vinceti et al.^134^	1 μg/L	0	1	8	0	0
	uranium	MCL	30 μg/L	0	0	0	0	1
		MCLG	0 μg/L	2	0	15	3	4
organic	atrazine	MCL(MCLG)	3 μg/L	0	0	0	0	0
		EU Directive	0.1 μg/L	7	0	0	0	0
	benzene	MCLG	0 μg/L	0	0	1	0	0
	bromodichloromethane	MCLG	0 μg/L	9	12	0	0	0
	dichloromethane	MCLG	0 μg/L	2	0	0	0	0
	1,2-dichloropropane	MCLG	0 μg/L	0	0	0	1	0
	tribromomethane	MCLG	0 μg/L	0	9	0	0	0
	TCE	MCLG	0 μg/L	0	0	1	0	0
	vinyl chloride	MCLG	0 μg/L	1	0	0	0	0

a MCL, maximum contaminant level;^97^ MCLG, maximum contaminant level goal;^97^ AL, technology treatment action level;^97^ DWHA, drinking water health advisory (lifetime);^97^ Vinceti et al.;^134^ EU Directive, European Union Directive.^131^ “MCL(MCLG)” indicates the same value for both.

### 3.3 TW Manganese

No Mn MCL^97^ (or WHO GV^89^) currently exists, but USEPA maintains a 300 μg L^–1^ lifetime drinking-water health advisory (DWHA; assumes 100% exposure from drinking water).^97^ Approximately 32% (18/56) of the samples in this study (75% in community C) exceeded or equaled the USEPA DWHA, raising concerns about prolonged exposures. More concerning, a Mn concentration greater than the 1 day acute exposure level of 1 mg L^–1^ for small (≤10 kg) children^97^ co-occurred in a private-well TW sample with a greater than MCL-equivalent U concentration, again emphasizing the elevated risk of unrecognized exposures in private-well TW. Community B Mn concentrations differed by an order of magnitude (*p* = 0.0002) between surface-water-sourced public-supply (median: 2 μg L^–1^) and groundwater-sourced (private-/public-supply median: 23 μg L^–1^) samples. Median Mn concentrations were more than an order of magnitude higher in community C samples (all groundwater-sourced), with no difference (*p* = 0.21) between private- and public-supply locations (medians 575 and 554 μg L^–1^, respectively). However, the highest Mn concentration in this study (2880 μg L^–1^) was observed in a private-well TW sample in community C, which also had the study’s highest dissolved Fe (4640 μg L^–1^) and only >MCL-equivalent U concentration (34 μg L^–1^). As noted above, frequent co-occurrence in groundwater-sourced TW samples of redox-reactive inorganics, including those (e.g., As and U) mobilized under different redox conditions, is consistent with widespread well-water redox heterogeneity.^34^

Across the US, approximately 6.9% of drinking-water-aquifer samples (*n* = 3662) exceeded 300 μg L^–1^ Mn, an exceedance rate comparable to those for MCL-equivalent concentrations of NO_3_–N (4.1%) and As (6.7%) in the same wells.^6^ Groundwater Mn concentrations in excess of 300 μg L^–1^ were associated with proximity to surface waters and organic-rich soils,^120^ consistent with the elevated Mn concentrations observed in community C, near a large regional lake. Growing concerns for cognitive, neurodevelopmental, and behavioral effects of long-term exposures in children have prompted calls to re-evaluate the regulation/monitoring of drinking-water Mn.^121,122^ To protect against neurological effects in bottle-fed infants, WHO^123^ recently released a provisional Mn GV of 80 μg L^–1^, a value exceeded by 39% (22/56) of samples in the current study (85% of community C).

### 3.4 TW Selenium

Elevated drinking-water Se has been proposed as a risk factor for adverse health outcomes,^124^ including amyotrophic lateral sclerosis (ALS), Parkinson’s disease,^125^ neurotoxicity,^126^ and skin cancer.^127^ Elevated serum Se concentrations have been associated with diabetes and elevated fasting glucose.^128,129^ While the USEPA Se MCLG is currently 50 μg L^–1^,^97,130^ growing concerns prompted a recent 20 μg L^–1^ European Commission parametric value for Se.^131^ Based on a comparative cohort (exposed, unexposed) study of long-term exposure to drinking-water Se in the range of 7–10 μg L^–1^ in northern Italy,^132,133^ a drinking-water limit of 1 μg L^–1^ was proposed to decrease the risk of adverse health effects, including neoplasms and endocrine and neurological diseases.^134^ In the current study, Se was detected in excess of 1 μg L^–1^ in 26% (9/34) of private-well TW samples (8/14 community A), but not in any public-supply samples.

### 3.5 TW Fluoride

Detected F concentrations (Figure S1, Table S3) were well below the USEPA MCL established to protect against bone fragility and skeletal fluorosis,^7,8^ indicating little concern for this toxic effect from TW exposures within the study area. However, all F concentrations in the current study were below the US Public Health Service^135^ optimum of 0.7 mg L^–1^ to prevent dental caries (Figure S1), consistent with groundwater results^6,136^ and dental-health concerns for children on private-wells^11^ across the US. The American Academy of Pediatrics^137^ and the Centers for Disease Control (CDC)^138^ recommend F supplementation for children if their drinking water contains <0.6 mg L^–1^ F.

### 3.6 TW Organics

**Figure 5:**
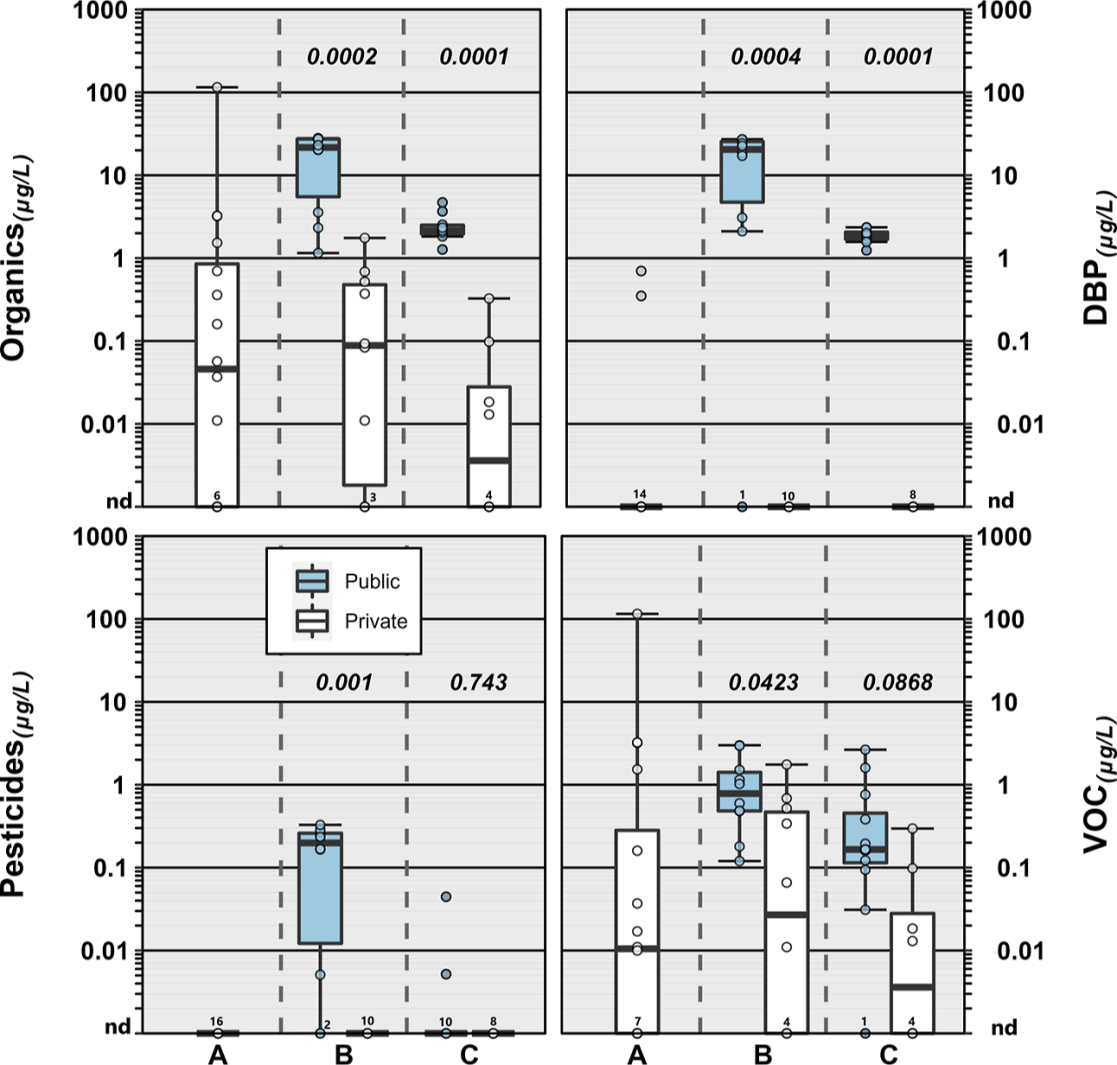
Cumulative concentrations (circles, ●) of all organics (plot A) and select organic classes (plots B–D) detected during 2019 in North Dakota and South Dakota in public-supply (shaded) and private-supply (unshaded) TW samples within each study area. Boxes, centerlines, and whiskers indicate interquartile range, median, and 5th and 95th percentiles, respectively. Numbers above each boxplot pair indicate the permuted probability that the centroids and dispersions are the same (PERMANOVA; 9999 permutations). “nd” indicates not detected.

TW samples were screened for 4 cyanotoxins, 22 DBP, 218 pesticide, 34 PFAS, 78 VOC, and 112 pharmaceutical analytes. Among the 63 organic analytes detected at least once, 59% (38) were detected in 5% (≤3) or fewer samples, with 20 (31%) detected only once. At least one organic analyte was detected in 79% (44/56) of the TW sample locations, with more than one detected in 59% (33/56) of locations. Higher numbers and cumulative concentrations of detected organics were observed in public-supply TW samples than in private supply in communities B and C (*p* = 0.0001), attributable primarily to DBP. In general, few organics were detected in private-well samples (Figures 4 and 5), with 35% (12/34) having no organic detections; a median detection of 1 for communities A (range: 1–9), B (range: nd–4), and C (range: nd–6); and no systematic detections. However, the highest individual (isopropyl alcohol; Chemical Abstract Service [CAS] number: 67-63-0) and cumulative concentrations of detected organics (all VOC) were observed in a private-well TW sample from community A; isopropyl alcohol is a common household solvent and personal care product.^139^

The VOC class dominated organic detections and concentrations in private-supply TW, on average (median) accounting for 100% of cumulative detections (IQR: 50–100%) and concentrations (IQR: 82–100%) in samples with detectable organics. The most frequent organic analyte detections in private-well TW (24%) were generally trace levels of 1,1-difluoroethane (CAS: 75-37-6), most commonly used as an aerosol propellant;^139^ similar frequency (27%) sporadic detections in public-supply samples are most readily reconciled with trace residential air contamination. The co-occurrence of multiple petroleum-hydrocarbon contaminants in two remote rural, private-well TW locations is consistent with groundwater contamination from farm-related refueling activities. VOC remained a frequently detected class in public-supply TW locations, with predominantly xylenes observed in community C and xylenes and the fuel-oxygenate, *t*-butyl alcohol, systematically detected in community B. However, consistent with previous findings,^29,30,43,44^ DBP dominated public supply organics, representing on average (median) 58% of detections and 92% of cumulative concentrations. Cumulative DBP concentrations were more than an order of magnitude higher (*p* < 0.0001) in surface-water-sourced public-supply TW samples (community B only; median: 24.2 μg L^–1^) than in groundwater-sourced public-supply locations (median: 2.0 μg L^–1^) in communities B and C. Consistent with widespread agricultural use within Corn Belt drainage basins,^140,141^ including the Missouri River basin,^142^ pesticides (median: 6) were detected (concentrations ≤ 0.33 μg L^–1^) in every surface-water-sourced public-supply sample but were rarely detected in any groundwater-sourced location in this study. Other notable, albeit less frequent, organic detections of growing human-health concerns included PFAS compounds (PFBA, one private supply, 83.7 ng L^–1^; PFBS, two private supplies, maximum 13.2 ng L^–1^) and cyanotoxins (cylindrospermopsin, one public supply, 90 ng L^–1^; saxitoxins, two private supplies, both 30 ng L^–1^), all below current lowest state drinking-water ALs or advisories for PFBA (7000 ng L^–1^),^143^ PFBS (345 ng L^–1^),^144^ cylindrospermopsin (700 ng L^–1^),^145^ and saxitoxins (300 ng L^–1^).^145^

Among the 63 detected organics, 16 have MCLG; among these 12 have individual MCL and 4 are addressed as a class by the trihalomethane (THM) MCL. No MCL or MCL-equivalent concentration was exceeded in public- or private-supply samples, respectively. However, seven detected analytes have an MCLG of zero. Among these MCLG exceedances, only bromodichloromethane, tribromomethane, and dichloromethane were detected in more than one sample and only in treated public supplies. The remaining MCLG (zero) exceedances were single detections each of 1,2-dichloropropane, benzene, trichloroethene (TCE), and vinyl chloride.

### 3.7 TW In Vitro ER, AR, and GR Bioactivity

While estrogenic activity was detected in five samples above the T47D-KBluc minimum detectable concentration [MDC; 0.0683 ng 17β-estradiol equivalents (E2Eq) L^–1^], and androgenic activity was detected in one sample above the CV1-chAR MDC (0.9 ng dihydrotestosterone equiv L^–1^), glucocorticoid activity was not detected above its bioassay MDC (Table S8). No detected activity (estrogen or androgen activity) exceeded respective drinking water effect-based trigger values (indicative of adverse health effects) previously developed for similar molecular-endpoint bioassays.^146^

### 3.8 TW Aggregated Screening Assessment: Σ_EAR_ and Σ_TQ_

We screened for TW cumulative-exposure effects of potential human-health interest using two analogous bioactivity-weighted approaches (Σ_EAR_, Σ_TQ_) with distinct strengths and limitations. Both are based on detected TW constituents and constrained by the analytical scope (468 organics and 33 inorganics), which, while extensive, is an orders-of-magnitude underestimate of the contaminant exposures documented in drinking-water sources. Likewise, both approaches are limited to available weighting factors (ToxCast ACC and human-health benchmarks, respectively) and assume cumulative effects are reasonably approximated by concentration addition.^83,85,147^ The Σ_EAR_ approach^29,48^ leverages high-throughput exposure-effects data for the 10,000 + organics and approximately 1000 vertebrate-cell-line molecular endpoints^148,149^ in the invitroDBv3.2 release^150^ of the ToxCast database to estimate potential cumulative activity at sensitive and possibly more protective sublethal molecular endpoints but has limited to no coverage of inorganic contaminants and unknown transferability to organ/organism scales.^151^ Importantly, the approach employed here aggregates contaminant bioactivity ratios across all endpoints without restriction to recognized modes of action as a precautionary screening for further investigation of potential effects but may not accurately reflect the apical effects that typically drive regulatory risk assessments.^48,151^ In contrast, the Σ_TQ_ HI approach provides insight into potential effects of simultaneous inorganic and organic exposures and is targeted at apical human-health effects, but it is notably constrained to available regulatory risk determinations.

**Figure 6:**
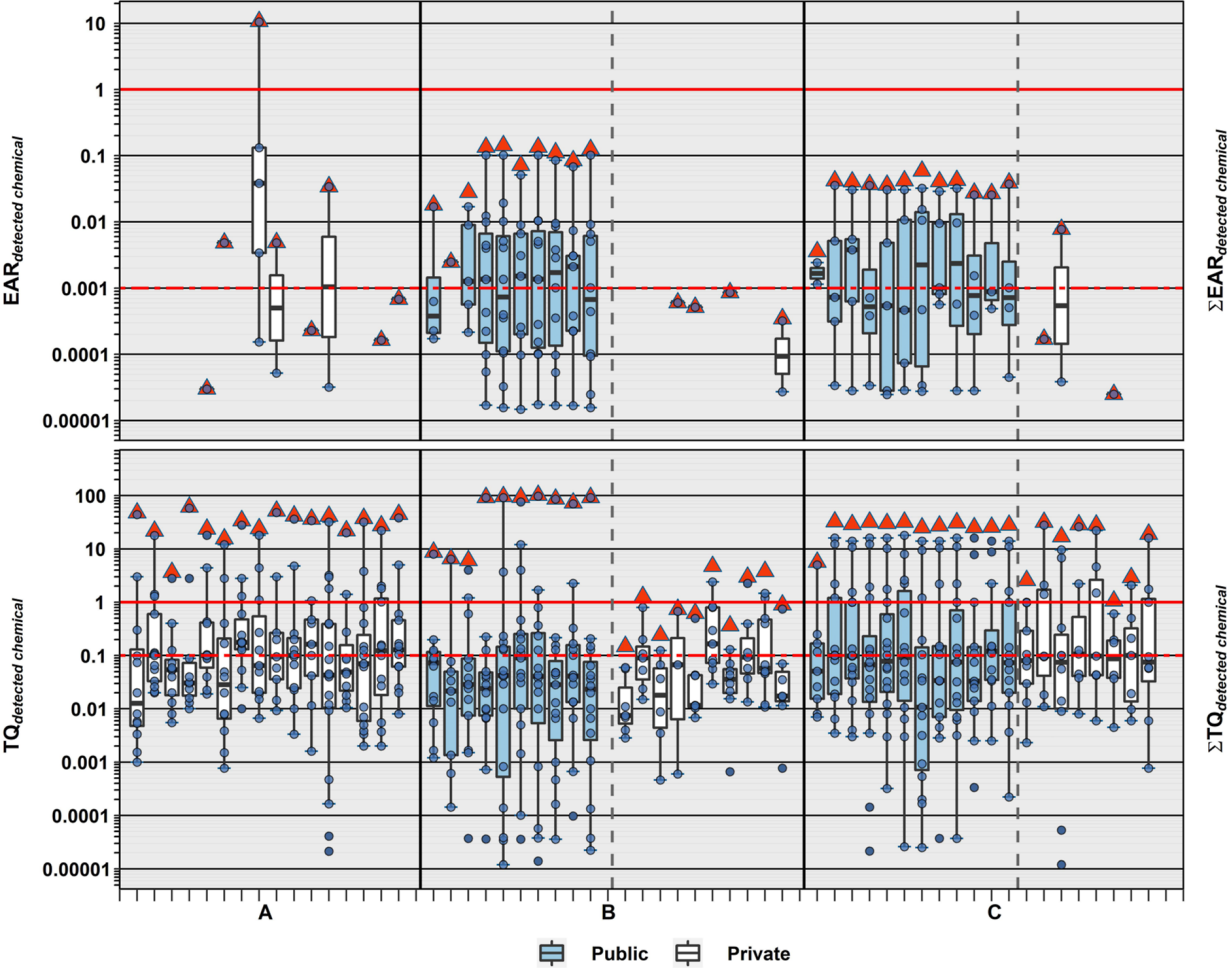
Top. Individual EAR values (circles, ●) and cumulative EAR (Σ_EAR_, sum of all detected; red triangles, ▲) across all assays for 32 organic analytes listed in ToxCast and detected in public-supply (shaded) and private-supply (unshaded) TW samples collected during 2019 in North Dakota and South Dakota. Solid and dashed red lines indicate concentrations shown to modulate effects in vitro and effects-screening-level thresholds (EAR = 0.001), respectively. Bottom. Human health benchmark-based individual TQ values (circles, ●) and cumulative TQ (Σ_TQ_, sum of all detected; red triangles, ▲) for inorganic and organic analytes listed in Table S12 and detected in treated public-supply (shaded) and untreated private-supply (unshaded) TW samples. Solid and dashed red lines indicate benchmark equivalent concentrations and effects-screening-level threshold of concern (TQ = 0.1), respectively. Boxes, centerlines, and whiskers indicate interquartile range, median, and 5th and 95th percentiles, respectively, for both plots.

Only about half (32) of the 63 organics detected in TW in this study and only 5 of 12 detected DBP had exact Chemical Abstract Services (CAS) number matches in the ToxCast invitroDBv3.2 database (Figure S2; Table S10). Consistent with the presence of DBP in public supply and the generally infrequent and low-concentration detections of organics in private supplies, site-specific Σ_EAR_ was higher (*p* = 0.0001) in public-supply TW (median: 0.0412) than in private supply (median: <0.00001) (Figure 6; Table S10). However, the highest EAR (and Σ_EAR_) in this study was observed in a private-well TW sample from community A with a concentration of 1-butanol more than 10 times that (ACC) shown to modulate molecular targets in vitro (i.e., solid red Σ_EAR_ = 1 line). Individual EAR or Σ_EAR_ near or above 0.1 in the seven surface-water-sourced TW samples from community B indicated an elevated probability of effects, driven primarily by the DBP, chlorodibromomethane. Exceedance of Σ_EAR_ = 0.001 (precautionary screening-level threshold of interest) in all public-supply and five private-supply samples indicated that further investigation of the cumulative biological activity from SHWS area TW exposures is warranted, even when considering only the organic contaminants detected in this study.

**Figure 7:**
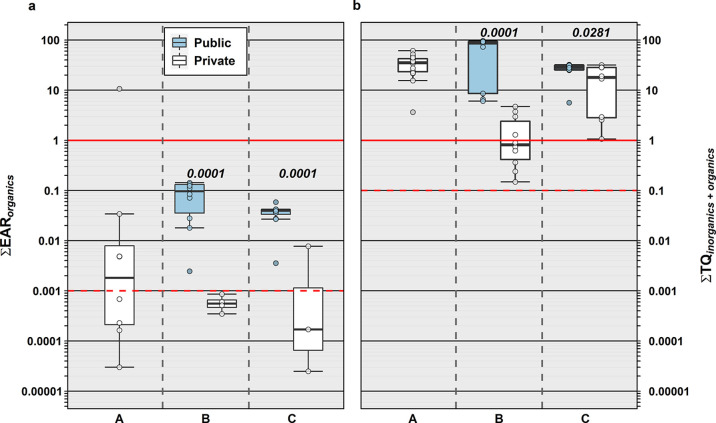
Cumulative EAR (Σ_EAR_, left, organics only) and cumulative TQ (Σ_TQ_, right, inorganics and organics) for analytes detected in public-supply (shaded) and private-supply (unshaded) TW samples during 2019 in North Dakota and South Dakota within each study area. Solid and dashed red lines indicate in vitro effect or benchmark equivalent exposures (Σ_EAR_ = 1, Σ_TQ_ = 1) and effects-screening-level thresholds of concern (Σ_EAR_ = 0.001, Σ_TQ_ = 0.1), respectively. Boxes, centerlines, and whiskers indicate interquartile range, median, and 5th and 95th percentiles, respectively. Numbers above each boxplot pair indicate the permuted probability that the centroids and dispersions are the same (PERMANOVA; 9999 permutations).

Every TW sample exceeded the Σ_TQ_ = 0.1 HI screening threshold of concern and all, but four, private-well TW samples exceeded Σ_TQ_ = 1 (Table S12). These Σ_TQ_ results indicate high probabilities of aggregated risks in SHWS private- and public-supply TW samples when considering exposures to both organic and inorganic chemicals (Figures 6 and S3; Table S13). Σ_TQ_ was driven primarily by inorganics (As, Mn, Pb, and U) in private-well TW samples and by organics (DBP) and inorganics (As, Mn, Pb, and U) in public-supply TW samples. Site-specific HI were higher (*p* = 0.0009) in public-supply (median: 30.6) than in private-supply (median: 20.3) TW (Figure 7), with only modest differences (*p* = 0.0301) in community C but approximately two orders-of-magnitude higher (*p* = 0.0001) median Σ_TQ_ in community B public-supply (median: 89.6) versus private-supply (median: 0.82) samples. Common-place DBP-driven exceedance of HI screening-levels of concern in public-supply TW, in this study and previously,^29,30,43,44^ reemphasize the public-health tradeoff of chlorine disinfection,^152,153^ the importance of better understanding of the cumulative DBP health risks,^152,154^ and the need for improved DBP-precursor (e.g., natural organic matter) removal prior to disinfection.^155,156^ Likewise, frequent exceedances of the Σ_TQ_ HI screening level of 0.1 in unregulated and generally unmonitored private-supply TW in this and previous studies^29,30,44^ reiterate the inherent human-health challenge of unmonitored TW.^6,12,21−23^ Simultaneous co-occurring inorganic and organic exposures and corresponding potentials for cumulative effects add weight to previous recommendations for systematic private-supply monitoring,^23^ with an analytical scope that more realistically reflects the breadth of inorganic and organic environmental contamination.^5,157^

### 3.9 Implications for Drinking-Water Treatment and TW Exposure Mitigation

The results indicate effective treatment of regulated contaminant exposures to below MCL levels in all public-supply TW samples in this study. Considering the multiple exceedances of health-only MCLG,^7,8^ however, complete communication of public-supply TW monitoring results, including exposures below current MCL, is needed to support consumer POU-treatment decision-making. Likewise, the ongoing SHWS As intervention has documented an effective reduction of TW As exposures in private-well locations in the study area using POU adsorptive media.^41^ In light of growing evidence for human-health effects of exposures to currently unregulated TW contaminants (e.g., unregulated DBP^154^ and PFAS^158^) or to regulated contaminants at <MCL concentrations (e.g., As^98^ and NO_3_^116^), common co-occurrences of multiple analytes with human-health implications in both private- and public-supply samples, including co-occurring exceedances of MCLG and Σ_TQ_ > 1, may reasonably raise consumer concerns^1,2^ and corresponding interest in POE/POU treatment.^159^ The median per sample number of health-benchmark exceedances in this study was two (range: 0–5), illustrating the importance of identifying stand-alone POE/POU treatment options for unregulated private-well TW, which are effective against multiple contaminants,^159,160^ and highlighting the potential value of POU treatment of public-supply TW for additional contaminant removal, including DBP.^159^

Several POE/POU treatment technologies are effective in reducing TW exposures to the contaminants identified in this study.^159^ The protectiveness of POE/POU approaches depends on the selection of appropriate filtration technologies for exposures of concern, timely and effective maintenance, and monitoring to confirm acceptable performance. For locations in the study area where geogenic contaminants like As and U are the only exposures of apparent concern, single-stage configurations of multiple treatment technologies are appropriate, including solid-block activated carbon, ion exchange media, redox media, and reverse osmosis (RO).^159,161^ However, broadly effective single-stage treatment technologies, such as RO, or multistage/multifiltration systems (sediment filter, redox media, activated carbon, ion exchange, RO, and UV disinfection) may be more appropriate for those locations with mixtures of inorganic and organic contaminants or unknown contaminant-exposure profiles (i.e., unmonitored private wells).^159^

## 4 Conclusions

Assessment and communication of TW contaminant exposures are essential to contaminant-risk-management and public-health decision-making at household and community scales. The perceived risks of and resultant resource commitments to drinking-water contaminant exposures are highly variable at both scales due to differences in availability of actionable drinking-water contaminant exposure/risk information, risk acceptability/tolerance thresholds, and overall exposure/risk portfolios (exposome).^153,162^ In the US, SDWA-stipulated annual public-supply consumer confidence reports support community and household-level decision-making, but decisions concerning additional community- or household-level treatment of public-supply TW are constrained by limited information on unregulated contaminants and on <MCL (or <AL) concentrations of regulated contaminants. Limited financial resources, water-quality expertise, and contaminant exposure data at the household scale fundamentally constrain private-supply decision-making and risk-mitigation options.^23,29,44^ Analytically extensive datasets like this study, which are intended to inform scientific and public-health understanding of the role of drinking water as a vector for human contaminant exposures and associated human-health outcomes, remain limited because broad assessment of regulated and unregulated contaminants are not generally conducted at the TW point of exposure in the US or worldwide.

These results indicate that simultaneous exposures to contaminants of human-health interest are common in both public- and private-supply TW locations across the study area. The bioactivity-weighted screening (Σ_EAR_ and Σ_TQ_) results (Tables S10 and S13) indicate that exposures to DBP and inorganics at <MCL concentrations are the primary drivers of human-health interest in public-supply TW in communities B and C. These public-supply results align with the previous findings^29,30,43,44^ and support the need for better understanding of exposure-effect relations and cumulative health risks of regulated/unregulated DBP^152,154^ and for improved source-water pretreatment technologies to address DBP precursors, such as surface-water natural organic matter.^156,163^ Exposures to As and U in groundwater-sourced drinking-water supplies have been reported previously in the study area and the concentrations observed in public-supply TW herein were below corresponding NPDWR MCL. However, because there is no known safe level of exposure for either compound (i.e., MCLG zero), community and individual interests in improved drinking-water facility processes and household treatment options are reasonable. The benchmark-weighted screening (Σ_TQ_) results (Table S13) also indicate that exposures to inorganics often at concentrations above NPDWR MCL-equivalent concentrations are frequent drivers of potential human-health concerns for private-supply TW across the study area, along with more sporadic detections of Pb and other anthropogenic contaminants.

Thus, these results substantiate continued expansion and connection of public-supply systems to limit contaminant exposures to <MCL concentrations and incorporation of well-maintained POE/POU treatment as an integral complementary line of consumer protection for public-supply TW during normal and outbreak conditions^29,161,164^ and as prudent protection against unrecognized simultaneous exposures to multiple contaminants in private-supply homes.^29,41^ These results emphasize the importance of continued characterization of POU TW exposures, especially in unregulated and unmonitored private supplies and small community water supplies, using an analytical coverage that serves as a realistic indicator of the breadth and complexity of inorganic and organic contaminant mixtures known to occur in ambient source waters^5,157^ to support models of TW contaminant exposures and related risks. Increased availability of such health-based monitoring data, including results below current, technically/economically constrained enforceable standards (e.g., MCL), is important to support public engagement in source-water protection and drinking-water treatment and to inform consumer POE/POU treatment decisions in these Tribal communities and throughout the US.

## Supporting Information Available

Site information; compound information for analyses performed by various laboratories for the USGS; parameters and concentrations of analyzed major anions and cations; detected organic compounds; inorganic analyte detections; concentrations of the analytes detected in organic analyte quality assurance field blanks; surrogate recovery or internal dilution standard summary statistics; mammalian bioactivities; endpoint combinations excluded from ToxCast evaluation; site-specific EAR; and human-health-based benchmarks; and site-specific TQ (XLSX)
Analytical methods, risk assessment, acknowledgments, and references (PDF)
