# Supplementary material for: Tapwater Exposures, Effects Potential, and Residential Risk Management in Northern Plains Nations
Source: ACS ES T Water. 2022 Sep 26;2(10):1772–88. doi: 10.1021/acsestwater.2c00293 (PMC9578051; doi:10.1021/acsestwater.2c00293)
Supplement: Supplementary file 2 — ew2c00293_si_002.pdf [file ew2c00293_si_002.pdf]

Tapwater Exposures, Effects Potential, and Residential Risk Management in Northern Plains Nations

Paul M. Bradley,[1] Kristin M. Romanok,[2] Kelly L. Smalling,[2] Michael J. Focazio,[3] Robert Charboneau,[4] Christine George,[5] Ana Navas-Acien,[6] Marcia O'Leary,[7] Reno Red Cloud,[8] Tracy Zacher,[7] Mary C. Cardon,[9] Christa Cuny,[7] Guthrie Ducheneaux,[7] Kendra Enright,[7] Nicola Evans,[9] James L. Gray,[10] David E. Harvey,[11] Michelle L. Hladik,[12] Leslie K. Kanagy,[10] Keith A. Loftin,[13] Richard Blaine McCleskey,[14] Elizabeth K. Medlock-Kakaley,[9] Shannon Meppelink,[15] Joshua F. Valder,[16] Christopher P. Weis[17]

[1]U.S. Geological Survey, Columbia, South Carolina, USA; [2]U.S. Geological Survey, Lawrenceville, New Jersey, USA; [3]U.S. Geological Survey, Reston, Virginia, USA; [4]Spirit Lake Tribe Office of Environmental Health, Fort Totten, North Dakota, USA; [5]Johns Hopkins Bloomberg School of Public Health, Baltimore, Maryland, USA; [6]Columbia Mailman School of Public Health, New York, New York, USA; [7]Missouri Breaks Industries Research Inc., Eagle Butte, South Dakota, USA; [8]Oglala Sioux Tribe Natural Resources Regulatory Agency, Pine Ridge, South Dakota, USA; [9]U.S. Environmental Protection Agency, Durham, North Carolina, USA; [10]U.S. Geological Survey, Lakewood, Colorado, USA; [11]Indian Health Service/HHS, Rockville, Maryland, USA; [12]U.S. Geological Survey, Sacramento, California, USA; [13]U.S. Geological Survey, Lawrence, Kansas, USA; [14]U.S. Geological Survey, Iowa City, Iowa, USA; [15]U.S. Geological Survey, Boulder, Colorado, USA; [16]U.S. Geological Survey, Rapid City, South Dakota, USA; [17]National Institute of Environmental Health Sciences/NIH, Bethesda, Maryland, USA

[*]Corresponding Author: Paul M. Bradley, USGS, South Atlantic Water Science Center, Columbia, SC 29210, pbradley@usgs.gov

The authors declare they have no actual or potential competing financial interests.

Methods

Analytical Methods

Tapwater (TW) samples were analyzed by United States Geological Survey (USGS) using 7 organic (6 classes; 468 unique analytes), 5 inorganic (34 ions/trace elements), and 2 field methods (Table S2), as discussed[1-4] and described in detail previously.[5-17] Organic analyte classes included cyanotoxin, disinfection byproduct(s) (DBP), pesticide, per/polyfluoroalkyl substance(s) (PFAS), volatile organic compound(s) (VOC), and pharmaceutical classes.

Per/poly-fluoroalkyl substances (PFAS) method details are provided in Kolpin et al.[17] Supporting Information. Pharmaceutical and pesticide samples were syringe filtered (0.7 µm nominal pore size, glass fiber) in the field. Pharmaceutical and VOC bottles were pretreated with ascorbic acid to neutralize chlorine/chloramine.

In vitro estrogen (ER), androgen (AR), and glucocorticoid (GR) bioactivities were assessed by the United States Environmental Protection Agency (USEPA) using the T47D-KBluc cell line (American Type Cell Culture, Manassas, Virginia; ATCC CRL-2865; human estrogen receptor α/β)[18,19] and the CV1 cell line (ATCC CCL-70) transduced (adenovirus) with the chimpanzee androgen receptor[20,21] or the human glucocorticoid receptor,[22] as described previously.[22-24] Cells for bioassays were plated in 96-well luminometer plates (T47D-KBluc: Greiner, Bio-One North America, Monroe, NC, CV1-hGR/chAR: Costar 3610, Corning Inc., Corning, NY) and standards, controls, and samples were run in quadruplicate, and each sample screen was at least duplicated. After 24 hr *in vitro* exposure, cells were visually scored for cytotoxicity and any wells with cells exhibiting cytotoxic effects were excluded from subsequent analysis.[22,25] Luminescence was quantified using a CLARIOstar luminometer (BMG Labtech, Cary, NC)[24] Endocrine-active samples were identified using a tiered screening process for tapwater.[26]

Biological equivalency values (BioEq) were calculated using an enrichment factor (EF) of 10,000.[27] BioEq above the respective assay minimum detectable concentration (T47KBluc: 0.068 ng 17β-Estradiol equivalents (Eq)/L; CV1-chAR: 0.9 ng 4,5α-Dihydrotestosterone Eq/L; and CV1-hGR: 5.41 ng Dexamethasone Eq/L) were considered positive for endocrine activity.[24,26] All results are in Tables S3-S4 and S8 and in Romanok et al.[28,29]

Risk Assessments

A screening-level assessment[30,31] of potential cumulative biological activity of mixed-organic contaminants in each TW sample was conducted as described.[2,32,33] The toxEval version 1.2.0 package[34] of the open source statistical software R[35] was used to sum (non-interactive concentration addition model[36-38] individual EAR (ratio of the detected concentration to the activity concentration at cutoff (ACC) from the Toxicity ForeCaster (ToxCast)[39] high-throughput screening data[40,41]) to estimate sample-specific cumulative EAR ($\sum_{EAR}$).[2,3,33] ACC estimates the point of departure concentration at which a defined threshold of response (cutoff) is achieved for a given biological activity and is less prone to violations of relative potency assumptions; for discussion, see.[33] ACC data in the toxEval v1.2.0 employed in the present study were from the August 2020 invitroDBv3.2 release of the ToxCast database.[40] Non-specific-endpoint, baseline, and unreliable response-curve assays were excluded.[4,33,42] $\sum_{EAR}$ results and exclusions are summarized in Tables S9-S11.

An analogous human-health-benchmark HI assessment[30,31,43] of the combined inorganic and organic contaminant risk also was conducted using toxEval v1.2.0[34] to sum the TQ (ratio of detected concentration to corresponding health-based benchmark) of individual detections to estimate sample-specific cumulative TQ ($\sum_{TQ}$).[44] A precautionary screening-level approach was employed based on the most protective human-health benchmark (i.e., lowest benchmark

concentration) among MCLG,[45,46] WHO Guideline Values (GV) and provisional GV (pGV),[47] USGS Health-Based Screening Level (HBSL),[48] and state drinking-water MCL or health advisories (DWHA). For the $\sum_{TQ}$ assessment, MCLG values of zero (i.e., no identified safe-exposure level for sensitive sub-populations, including infants, children, the elderly, and those with compromised immune systems and chronic diseases[46,49]) were set to the respective method reporting limit, except for Pb, which was set to 1 µg L^{-1} as suggested by the American Academy of Pediatrics.[50] $\sum_{TQ}$ results and respective health-based benchmarks are summarized in Tables S12-S13

Acknowledgements

We thank the Strong Heart Water Study participants and participating Tribal Nations for their support of this drinking-water research. This research was conducted and funded by the USGS Ecosystems Mission Area, Environmental Health Programs. Any use of trade, firm, or product names is for descriptive purposes only and does not imply endorsement by the U.S. Government. The findings and conclusions in this article do not necessarily represent the views or policies of the US Environmental Protection Agency, NIH/National Institute of Environmental Health Sciences, Indian Health Service, or Bureau of Indian Affairs. This report contains CAS Registry Numbers®, which is a registered trademark of the American Chemical Society. CAS recommends the verification of the CASRNs through CAS Client ServicesSM.

References

(1) Romanok, K. M.; Kolpin, D. W.; Meppelink, S. M.; Argos, M.; Brown, J.; DeVito, M.; Dietz, J. E.; C.E., G.; Gray, J.; Higgins, C. P.; Hladik, M. L.; Iwanowicz, L. R.; McCleskey, B. R.; McDonough, C.; Meyers, M. T.; Strynar, M.; Weis, C. P.; Wilson, V.; Bradley, P. M. *Methods used for the collection and analysis of chemical and biological data for the Tapwater Exposure Study, United States, 2016–17*; U.S. Geological Survey Open-File Report 2018-1098; Reston, VA, **2018**; p 81. https://doi.org/10.3133/ofr20181098

(2) Bradley, P. M.; Kolpin, D. W.; Romanok, K. M.; Smalling, K. L.; Focazio, M. J.; Brown, J. B.; Cardon, M. C.; Carpenter, K. D.; Corsi, S. R.; DeCicco, L. A.; Dietze, J. E.; Evans, N.; Furlong, E. T.; Givens, C. E.; Gray, J. L.; Griffin, D. W.; Higgins, C. P.; Hladik, M. L.; Iwanowicz, L. R.; Journey, C. A.; Kuivila, K. M.; Masoner, J. R.; McDonough, C. A.; Meyer, M. T.; Orlando, J. L.; Strynar, M. J.; Weis, C. P.; Wilson, V. S., Reconnaissance of mixed organic and inorganic chemicals in private and public supply tapwaters at selected residential and workplace sites in the United States. *Environ. Sci. Technol.* **2018**, DOI https://doi.org/10.1021/acs.est.8b04622.

(3) Bradley, P. M.; Argos, M.; Kolpin, D. W.; Meppelink, S. M.; Romanok, K. M.; Smalling, K. L.; Focazio, M. J.; Allen, J. M.; Dietze, J. E.; Devito, M. J.; Donovan, A. R.; Evans, N.; Givens, C. E.; Gray, J. L.; Higgins, C. P.; Hladik, M. L.; Iwanowicz, L. R.; Journey, C. A.; Lane, R. F.; Laughrey, Z. R.; Loftin, K. A.; McCleskey, R. B.; McDonough, C. A.; Medlock-Kakaley, E.; Meyer, M. T.; Putz, A. R.; Richardson, S. D.; Stark, A. E.; Weis, C. P.; Wilson, V. S.; Zehraoui, A., Mixed organic and inorganic tapwater exposures and potential effects in greater Chicago area, USA. *Sci. Total Environ.* **2020**, 137236, DOI https://doi.org/10.1016/j.scitotenv.2020.137236.

(4) Bradley, P. M.; LeBlanc, D. R.; Romanok, K. M.; Smalling, K. L.; Focazio, M. J.; Cardon, M. C.; Clark, J. M.; Conley, J. M.; Evans, N.; Givens, C. E.; Gray, J. L.; Earl Gray, L.; Hartig, P. C.; Higgins, C. P.; Hladik, M. L.; Iwanowicz, L. R.; Loftin, K. A.; Blaine McCleskey, R.; McDonough, C. A.; Medlock-Kakaley, E. K.; Weis, C. P.; Wilson, V. S., Public and private tapwater: Comparative analysis of contaminant exposure and potential risk, Cape Cod, Massachusetts, USA. *Environ. Int.* **2021,** *152*, 106487, DOI https://doi.org/10.1016/j.envint.2021.106487.

(5) Rose, D.; Sandstrom, M.; Murtagh, L. *Methods of the National Water Quality Laboratory. Chapter B12. Determination of heat purgeable and ambient purgeable volatile organic*

compounds in water by gas chromatography/mass spectrometry; U.S. Geological Survey Techniques and Methods. Book 5. Laboratory Analysis; **2016**; p 61. https://doi.org/10.3133/tm5B12

(6) Sandstrom, M. W.; Kanagy, L. K.; Anderson, C. A.; Kanagy, C. J. *Methods of the National Water Quality Laboratory. Chapter B11. Determination of pesticides and pesticide degradates in filtered water by direct aqueous-injection liquid chromatography-tandem mass spectrometry*; U.S. Geological Survey Techniques and Methods. Book 5. Laboratory Analysis; **2015**; p 73. https://doi.org/10.3133/tm5B11

(7) Hladik, M. L.; Focazio, M. J.; Engle, M., Discharges of produced waters from oil and gas extraction via wastewater treatment plants are sources of disinfection by-products to receiving streams. *Sci. Total Environ.* **2014,** *466*, 1085-1093, DOI https://doi.org/10.1016/j.scitotenv.2013.08.008.

(8) Furlong, E.; Noriega, M.; Kanagy, C.; Kanagy, L.; Coffey, L.; Burkhardt, M. *Methods of the National Water Quality Laboratory. Chapter B10. Determination of human-use pharmaceuticals in filtered water by direct aqueous injection–high-performance liquid chromatography/tandem mass spectrometry*; U.S. Geological Survey Techniques and Methods. Book 5. Laboratory Analysis. Chap. B10; **2014**; p 49. http://doi.org/10.3133/tm5B10

(9) Fishman, M. J.; Friedman, L. C. *Methods for determination of inorganic substances in water and fluvial sediments*; U.S. Geological Survey Techniques of Water-Resources Investigations 05-A1; **1989**; p 545. https://doi.org/10.3133/twri05A1

(10) Hoffman, G. L.; Fishman, M. J.; Garbarino, J. R. *Methods of Analysis by the US Geological Survey National Water Quality Laboratory: In-bottle Acid Digestion of Whole-water Samples*; U.S. Geological Survey Open-File Report 96-225; US Department of the Interior, US Geological Survey: **1996**; p 34. https://doi.org/10.3133/ofr96225

(11) Graham, J. L.; Loftin, K. A.; Meyer, M. T.; Ziegler, A. C., Cyanotoxin mixtures and taste-and-odor compounds in cyanobacterial blooms from the Midwestern United States. *Environ. Sci. Technol.* **2010,** *44*, (19), 7361-7368, DOI https://doi.org/10.1021/es1008938.

(12) Loftin, K. A.; Graham, J. L.; Hilborn, E. D.; Lehmann, S. C.; Meyer, M. T.; Dietze, J. E.; Griffith, C. B., Cyanotoxins in inland lakes of the United States: Occurrence and potential recreational health risks in the EPA National Lakes Assessment 2007. *Harmful Algae* **2016,** *56*, 77-90, DOI https://doi.org/10.1016/j.hal.2016.04.001.

(13) Pfaff, J. D. *Method 300.0, Determination of inorganic anions by ion chromatography. Revision 2.1*; USEPA Method 300.0; U.S. Environmental Protection Agency: **1993**; p 30.

(14) Ball, J. W.; McCleskey, R. B., A new cation-exchange method for accurate field speciation of hexavalent chromium. *Talanta* **2003,** *61*, (3), 305-313, DOI https://doi.org/10.1016/S0039-9140(03)00282-0.

(15) Hergenreder, R. L. *Trace Metals in Waters by GFAAS, in Accordance with U.S. EPA and Health Canada Requirements*; Perkin Elmer, Inc.: Waltham, MA, **2011**; p 5. https://www.perkinelmer.com/lab-solutions/resources/docs/PinAAcleTraceMetalsinWaterbyGFAAAppNote.pdf

(16) U.S. Environmental Protection Agency *Inductively coupled plasma-optical emission spectrometry, Method 6010D*; EPA SW-846 Update V, accessed November 2, 2017; **2014**; p 35. https://www.epa.gov/sites/production/files/2015-12/documents/6010d.pdf

(17) Kolpin, D. W.; Hubbard, L. E.; Cwiertny, D. M.; Meppelink, S. M.; Thompson, D. A.; Gray, J. L., A comprehensive statewide spatiotemporal stream assessment of per- and polyfluoroalkyl substances (PFAS) in an agricultural region of the United States. *Environ. Sci. Technol. Lett.* **2021,** *8*, (11), 981-988, DOI https://doi.org/10.1021/acs.estlett.1c00750.

(18) Wilson, V. S.; Bobseine, K.; Gray, L. E., Development and characterization of a cell line that stably expresses an estrogen-responsive luciferase reporter for the detection of estrogen receptor agonist and antagonists. *Toxicol. Sci.* **2004,** *81*, (1), 69-77, DOI https://doi.org/10.1093/toxsci/kfh180.

(19) Wilson, V. S.; Bobseine, K.; Lambright, C. R.; Gray, L. E., Jr., A novel cell line, MDA-kb2, that stably expresses an androgen- and glucocorticoid-responsive reporter for the detection of hormone receptor agonists and antagonists. *Toxicol. Sci.* **2002,** *66*, (1), 69-81, DOI https://doi.org/10.1093/toxsci/66.1.69.

(20) Hartig, P. C.; Bobseine, K. L.; Britt, B. H.; Cardon, M. C.; Lambright, C. R.; Wilson, V. S.; Gray, L. E., Jr., Development of two androgen receptor assays using adenoviral transduction of MMTV-Luc reporter and/or hAR for endocrine screening. *Toxicol. Sci.* **2002,** *66*, (1), 82-90, DOI https://doi.org/10.1093/toxsci/66.1.82.

(21) Hartig, P. C.; Cardon, M. C.; Lambright, C. R.; Bobseine, K. L.; Gray, L. E.; Wilson, V. S., Substitution of synthetic chimpanzee androgen receptor for human androgen receptor in competitive binding and transcriptional activation assays for EDC screening. *Toxicol. Lett.* **2007,** *174*, (1), 89-97, DOI https://doi.org/10.1016/j.toxlet.2007.08.013.

(22) Conley, J.; Evans, N.; Cardon, M.; Rosenblum, L.; Iwanowicz, L.; Hartig, P.; Schenck, K.; Bradley, P.; Wilson, V., Occurrence and in vitro bioactivity of estrogen, androgen, and glucocorticoid compounds in a nationwide screen of United States stream waters. *Environ. Sci. Technol.* **2017,** *51*, (9), 4781-4791, DOI http://doi.org/10.1021/acs.est.6b06515.

(23) Conley, J.; Evans, N.; Mash, H.; Rosenblum, L.; Schenck, K.; Glassmeyer, S.; Furlong, E.; Kolpin, D.; Wilson, V., Comparison of in vitro estrogenic activity and estrogen concentrations in source and treated waters from 25 US drinking water treatment plants. *Sci. Total Environ.* **2017,** *579*, 1610-1617, DOI https://doi.org/10.1016/j.scitotenv.2016.02.093.

(24) Medlock Kakaley, E. K.; Blackwell, B. R.; Cardon, M. C.; Conley, J. M.; Evans, N.; Feifarek, D. J.; Furlong, E. T.; Glassmeyer, S. T.; Gray, L. E.; Hartig, P. C.; Kolpin, D. W.; Mills, M. A.; Rosenblum, L.; Villeneuve, D. L.; Wilson, V. S., De Facto Water Reuse: Bioassay suite approach delivers depth and breadth in endocrine active compound detection. *Sci. Total Environ.* **2020,** *699*, 134297, DOI https://doi.org/10.1016/j.scitotenv.2019.134297.

(25) Bhatia, S. K.; Yetter, A. B., Correlation of visual in vitro cytotoxicity ratings of biomaterials with quantitative in vitro cell viability measurements. *Cell Biology and Toxicology* **2008,** *24*, (4), 315-319, DOI https://doi.org/10.1007/s10565-007-9040-z.

(26) Medlock Kakaley, E. K.; Cardon, M. C.; Evans, N.; Iwanowicz, L. R.; Allen, J. M.; Wagner, E.; Bokenkamp, K.; Richardson, S. D.; Plewa, M. J.; Bradley, P. M.; Romanok, K. M.; Kolpin, D. W.; Conley, J. M.; Gray Jr, L. E.; Hartig, P. C.; Wilson, V. S., In vitro effects-based method and water quality screening model for use in pre- and post-distribution treated waters. *Environ. Sci. Technol.* **2021**, DOI https://doi.org/10.1016/j.scitotenv.2020.144750.

(27) Escher, B.; Leusch, F., *Bioanalytical tools in water quality assessment.* IWA Publishing: 2011.

(28) Romanok, K. M.; Bradley, P. M. *Target-Chemical Concentration Results for Assessment of Mixed-Organic/Inorganic Chemical and Biological Exposures in North Dakota and South Dakota Tapwater, 2019*; U.S. Geological Survey data release https://doi.org/10.5066/P9KP2NP4; **2021**.

(29) Romanok, K. M.; Bradley, P. M.; McCleskey, R. B. *Inorganic Concentration Results for Assessment of Mixed-Organic/Inorganic Chemical and Biological Exposures in North Dakota and South Dakota Tapwater, 2019*; U.S. Geological Survey data release https://doi.org/10.5066/P9DBCKT4; **2021**.

(30) U.S. Environmental Protection Agency *EPA's National-scale Air Toxics Assessment, An Overview of Methods for EPA's National-Scale Air Toxics Assessment*; **2011**.

(31) Goumenou, M.; Tsatsakis, A., Proposing new approaches for the risk characterisation of single chemicals and chemical mixtures: The source related Hazard Quotient (HQS) and Hazard Index (HIS) and the adversity specific Hazard Index (HIA). *Toxicology Reports* **2019,** *6*, 632-636, DOI https://doi.org/10.1016/j.toxrep.2019.06.010.

(32) Bradley, P. M.; Journey, C. A.; Berninger, J. P.; Button, D. T.; Clark, J. M.; Corsi, S. R.; DeCicco, L. A.; Hopkins, K. G.; Huffman, B. J.; Nakagaki, N.; Norman, J. E.; Nowell, L. H.; Qi, S. L.; VanMetre, P. C.; Waite, I. R., Mixed-chemical exposure and predicted effects potential in wadeable southeastern USA streams. *Sci. Total Environ.* **2019,** *655*, 70-83, DOI https://doi.org/10.1016/j.scitotenv.2018.11.186.

(33) Blackwell, B. R.; Ankley, G. T.; Corsi, S. R.; De Cicco, L. A.; Houck, K. A.; Judson, R. S.; Li, S.; Martin, M. T.; Murphy, E.; Schroeder, A., An" EAR" on environmental surveillance and monitoring: A case study on the use of exposure-activity ratios (EARs) to prioritize sites, chemicals, and bioactivities of concern in Great Lakes waters. *Environ. Sci. Technol.* **2017,** *51*, (15), 8713-8724, DOI https://doi.org/10.1021/acs.est.7b01613.

(34) De Cicco, L.; Corsi, S. R.; Villeneuve, D.; Blackwell, B. R.; Ankley, G. T. toxEval: Evaluation of measured concentration data using the ToxCast high-throughput screening database or a user-defined set of concentration benchmarks. R package version 1.0.0. **2018**, (May 1, 2018)

(35) R Development Core Team *R: A Language and Environment for Statistical Computing. Version 3.5.2*; R Foundation for Statistical Computing: Vienna Austria, **2019**.

(36) Cedergreen, N.; Christensen, A. M.; Kamper, A.; Kudsk, P.; Mathiassen, S. K.; Streibig, J. C.; Sørensen, H., A review of independent action compared to concentration addition as reference models for mixtures of compounds with different molecular target sites. *Environ. Toxicol. Chem.* **2008,** *27*, (7), 1621-1632, DOI https://doi.org/10.1897/07-474.1.

(37) Altenburger, R.; Scholze, M.; Busch, W.; Escher, B. I.; Jakobs, G.; Krauss, M.; Krüger, J.; Neale, P. A.; Ait-Aissa, S.; Almeida, A. C., Mixture effects in samples of multiple contaminants–An inter-laboratory study with manifold bioassays. *Environ. Int.* **2018,** *114*, 95-106, DOI https://doi.org/10.1016/j.envint.2018.02.013.

(38) Stalter, D.; O'Malley, E.; von Gunten, U.; Escher, B. I., Mixture effects of drinking water disinfection by-products: implications for risk assessment. *Environmental Science: Water Research & Technology* **2020**, DOI https://doi.org/10.1039/C9EW00988D.

(39) U.S. Environmental Protection Agency, CompTox Chemicals Dashboard. **2020**, accessed January 22, 2020, at https://comptox.epa.gov/dashboard.

(40) U.S. Environmental Protection Agency, EPA's National Center for Computational Toxicology: ToxCast Database (invitroDB) vers.5.0. In 2020.

(41) U.S. Environmental Protection Agency ToxCast & Tox21 Summary Files from invitrodb_v3. **2019**, (July 5, 2019)

(42) Bradley, P. M.; Padilla, I. Y.; Romanok, K. M.; Smalling, K. L.; Focazio, M. J.; Breitmeyer, S. E.; Cardon, M. C.; Conley, J. M.; Evans, N.; Givens, C. E.; Gray, J. L.; Earl Gray, L.; Hartig, P. C.; Higgins, C. P.; Hladik, M. L.; Iwanowicz, L. R.; Lane, R. F.; Loftin, K. A.; Blaine McCleskey, R.; McDonough, C. A.; Medlock-Kakaley, E.; Meppelink, S.; Weis, C. P.; Wilson, V. S., Pilot-scale expanded assessment of inorganic and organic tapwater exposures and predicted effects in Puerto Rico, USA. *Sci. Total Environ.* **2021**, 147721, DOI https://doi.org/10.1016/j.scitotenv.2021.147721.

(43) U.S. Environmental Protection Agency *Sustainable Futures / Pollution Prevention (P2) Framework Manual*; EPA-748-B12-001; U.S. Environmental Protection Agency: Washington, D.C., **2012**; p 326. https://www.epa.gov/sustainable-futures/sustainable-futures-p2-framework-manual

(44) Corsi, S. R.; De Cicco, L. A.; Villeneuve, D. L.; Blackwell, B. R.; Fay, K. A.; Ankley, G. T.; Baldwin, A. K., Prioritizing chemicals of ecological concern in Great Lakes tributaries using high-throughput screening data and adverse outcome pathways. *Sci. Total Environ.* **2019,** *686*, 995-1009, DOI https://doi.org/10.1016/j.scitotenv.2019.05.457.

(45) U.S. Environmental Protection Agency, 40 C.F.R. § 131: Water Quality Standards. In *40 C.F.R. § 131*, U.S. Environmmental Protection Agency,: Washington, DC, 2017.

(46) U.S. Environmental Protection Agency National Primary Drinking Water Regulations. **2021**, (July 11, 2021)

(47) World Health Organization (WHO), *Guidelines for drinking-water quality, Fourth edition incorporating first addendum*. 2011; Vol. 38.

(48) Norman, J. E.; Toccalino, P. L.; Morman, S. A. Health-Based Screening Levels for evaluating water-quality data (2nd ed.). **2018**, https://doi.org/10.5066/F71C1TWP (February 10, 2020)

(49) U.S. Environmental Protection Agency How EPA Regulates Drinking Water Contaminants. **2021**, https://www.epa.gov/dwregdev/how-epa-regulates-drinking-water-contaminants (July 11, 2021)

(50) Lanphear, B.; Lowry, J.; Ahdoot, S.; Baum, C.; Bernstein, A.; Bole, A.; Brumberg, H.; Campbell, C.; Pacheco, S.; Spanier, A.; Trasande, L.; Osterhoudt, K.; Paulson, J.; Sandel, M.; Rogers, P., Prevention of childhood lead toxicity: Policy statement of the American Academy of Pediatrics Council on Environmental Health. *Pediatrics* **2016,** *138*, (1), e20161493, DOI https://doi.org/10.1542/peds.2016-1493.
